# Prenatal transplantation of human amniotic fluid stem cell could improve clinical outcome of type III spinal muscular atrophy in mice

**DOI:** 10.1038/s41598-021-88559-z

**Published:** 2021-04-28

**Authors:** Steven W. Shaw, Shao-Yu Peng, Ching-Chung Liang, Tzu-Yi Lin, Po-Jen Cheng, T’sang-T’ang Hsieh, Hao-Yu Chuang, Paolo De Coppi, Anna L. David

**Affiliations:** 1 College of Medicine, Chang Gung University, Taoyuan, 333 Taiwan; 2 Department of Obstetrics and Gynecology, Taipei Chang Gung Memorial Hospital, No. 199, Dun-Hua North Road, Taipei, 105 Taiwan; 3 Prenatal Cell and Gene Therapy Group, Elizabeth Garrett Anderson Institute for Women’s Health, University College London, London, WC1E 6HU UK; 4 Department of Animal Science, National Pingtung University of Science and Technology, Pingtung, 912 Taiwan; 5 Department of Obstetrics and Gynecology, Linkou Chang Gung Memorial Hospital, 333, Taoyuan, Taiwan; 6 Division of Neurosurgery, Tainan Municipal An-Nan Hospital, Tainan, 709 Taiwan; 7 Cell Therapy Center, Tainan Municipal An-Nan Hospital, Tainan, 709 Taiwan; 8 Stem Cells and Regenerative Medicine Section, Institute of Child Health, University College London, London, WC1N 1EH UK; 9 Great Ormond Street Hospital NHS Trust, London, WC1N 1EH UK

**Keywords:** Stem cells, Neurological disorders

## Abstract

Spinal muscular atrophy (SMA) is a single gene disorder affecting motor function in uterus. Amniotic fluid is an alternative source of stem cell to ameliorate SMA. Therefore, this study aims to examine the therapeutic potential of Human amniotic fluid stem cell (hAFSC) for SMA. Our SMA model mice were generated by deletion of exon 7 of Smn gene and knock-in of human *SMN2*. A total of 16 SMA model mice were injected with 1 × 10^5^ hAFSC in uterus, and the other 16 mice served as the negative control. Motor function was analyzed by three behavioral tests. Engraftment of hAFSC in organs were assessed by flow cytometry and RNA scope. Frequency of myocytes, neurons and innervated receptors were estimated by staining. With hAFSC transplantation, 15 fetuses survived (93.75% survival) and showed better performance in all motor function tests. Higher engraftment frequency were observed in muscle and liver. Besides, the muscle with hAFSC transplantation expressed much laminin α and PAX-7. Significantly higher frequency of myocytes, neurons and innervated receptors were observed. In our study, hAFSC engrafted on neuromuscular organs and improved cellular and behavioral outcomes of SMA model mice. This fetal therapy could preserve the time window and treat in the uterus.

## Introduction

Spinal muscular atrophy (SMA) affects about 8.5 to 10.3 of 100,000 newborns^1^. This is a life-threatening neurodegenerative disease with autosomal recessive inheritance. Congenital deletion or mutation of survival motor neuron (SMN) 1 gene on chromosome 5q13 leads to depletion of SMN protein^2^. Then, neurons from the anterior horn of spinal cord become too little to induce muscle contraction^3^. Besides, another similar gene, SMN2, is essential to the production of SMN protein as well. However, SMN2 could only compensate partially for the absence of SMN1 due to its incomplete version. The number of SMN2 copies is correlated to the severity of SMA. According to the gestational age of onset time and level of motor function, SMA was classified into three types. Type I SMA caused abnormal inactivity in the later stage of pregnancy and several challenges in fundamental function, including breathing and swallowing. Respiratory failure usually results in death at two years old. Type II SMA allowed normal development until 6 months old but no walking ability. These patients often lead to a longer life, about 25 years old. Type III SMA merely contributed to difficulty in climbing stairs and getting up from the floor, but the limitation of motion would progressively grow and require a wheelchair. However, their lifespan is approximately normal^4^. No therapy could treat SMA completely nowadays. However, type III SMA patients have milder symptoms and universally survive to an average age^5^. Therefore, several therapies were investigated to cure this type of disorder.

Certain stem cells have shown their ability to treat neuromuscular diseases. For instance, neural stem cells could differentiate into motor neurons and decrease the death of neuron in SMA murine model^6^. Embryonic stem cells demonstrated their potential of forming neuromuscular junction and differentiation into neuron to repair a musculocutaneous nerve in SMA rat^7^. Induced pluripotent stem cells were proved to delay the atrophy of muscle and retain neuromuscular function in SMA murine model^8^. In brief, these stem cells had the common potential of neural differentiation for treating neuromuscular diseases. By delivering neuroprotective factors and replacing degenerated cells, these stem cells could delay the progression of the disease and restore neuromuscular function^9,10^. Human amniotic fluid stem sell (hAFSC) shared the capability of differentiation and regeneration^11,12^. In previous studies, hAFSC held the promise to cure several diseases of the nervous system, including white matter disease and myelomeningocele^13,14^. Also, hAFSC transplantation could be combined with fetal surgery to be applied in mice with spinal bifida^15^. SMA represented another candidate disease to be treated prenatally with hAFSC transplantation in uterus^16^. With less cell count, irreversible damage from SMA could be prevented as soon as possible^17^.

To our knowledge, hAFSC has never been tested in SMA model as a potential therapeutic treatment prenatally. Prenatal diagnosis of SMA could be completed by amniocentesis between 15 and 20th gestational week. Simultaneously, stem cells could be harvested without controversial ethical issues^18,19^. Compared with postnatal therapy with NSC and iPSC transplantation, much less cell dose was required in prenatal therapy. Additionally, transplantation of hAFSC in early gestation could cease progression of pathophysiology and prevent irreversible damage as soon as possible in comparison with the administration of Nusinersen and Onasemnogene abeparvovec in the childhood. After the injection of hAFSC in uterus, the cells were able to migrate to several host organs^20^. Consequently, the survival of neurons and muscle could be elevated to improve the outcome of SMA. Besides, SMA mice model provided a useful in vivo system for studying the potential of hAFSC therapy. The objective of this study was to investigate the therapeutic effectiveness of hAFSC transplantation for SMA.

## Results

### Stem cell positive characteristics of hAFSC

**Figure 1:**
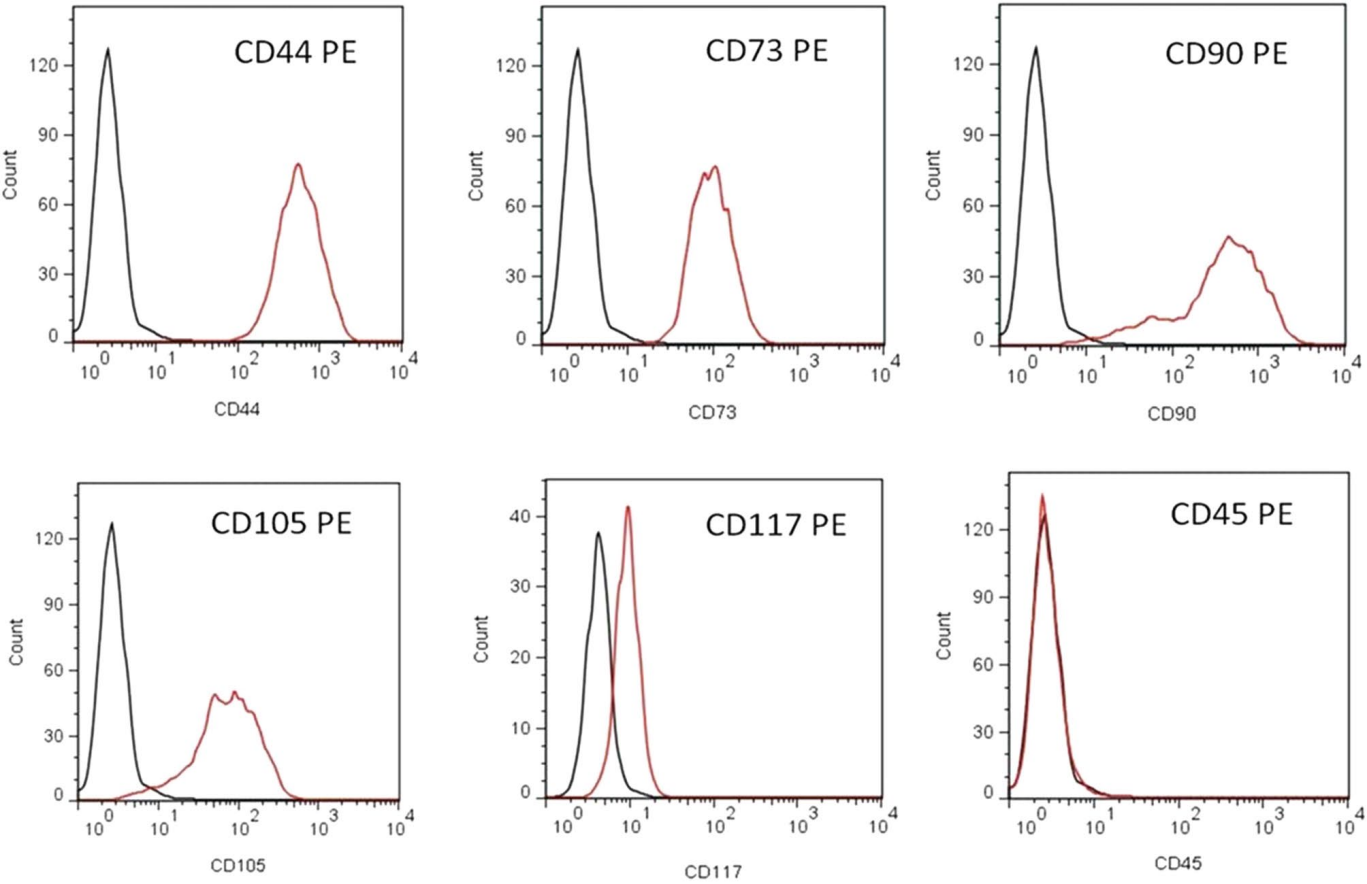
Flow cytometry analysis of surface marker of hAFSC. hAFSCs were positive for CD44, CD73, CD90, CD105 and CD117, markers of MSC, and negative for CD45, a marker of leukocyte. *hAFSC* human amniotic fluid stem cell, *MSC* mesenchymal stem cell, *PE* phycoerythrin.

**Figure 2:**
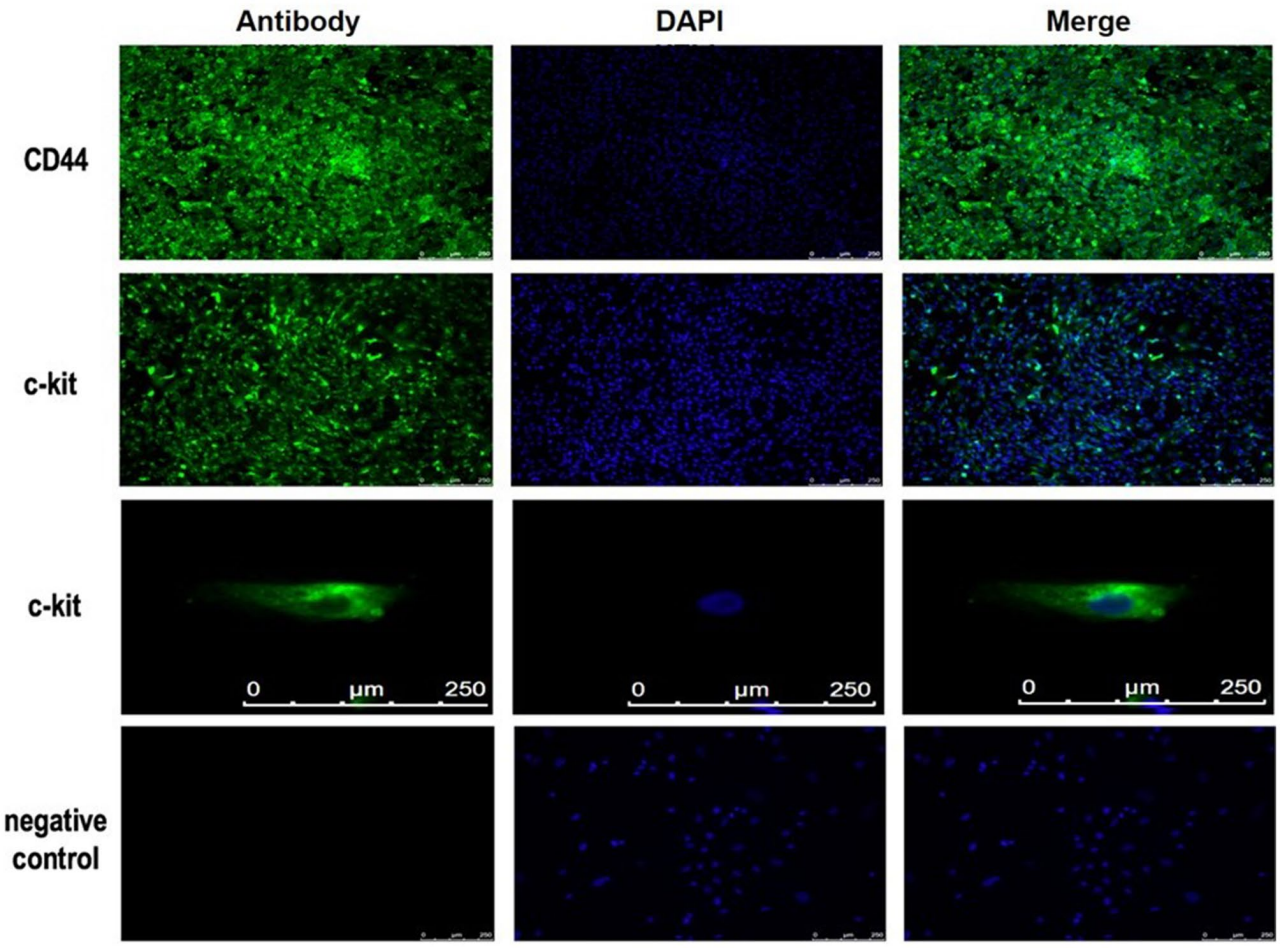
Immunocytochemistry staining of surface marker of hAFSC. hAFSC expressed enormous CD44 and c-kit, markers of stem cell. *hAFSC* human amniotic fluid stem cell.

Amniotic fluid was sampled by amniocentesis from pregnant women during 15th to 20th gestational week. Then, stem cells expressing MSC markers were isolated from amniotic fluid and cultured in an amniotic medium. By flow cytometry, these hAFSC were positive for several surface markers of MSC, such as CD44, CD73, CD90, CD105 and CD117 (Fig. 1). However, hAFSC were negative for CD45, an immune and hematopoietic marker. By immunocytochemistry staining for DAPI, the hAFSCs expressed enormous stem cell markers, including CD44 and c-kit (Fig. 2).

### Transplantation of hAFSC in SMA mice

**Figure 3:**
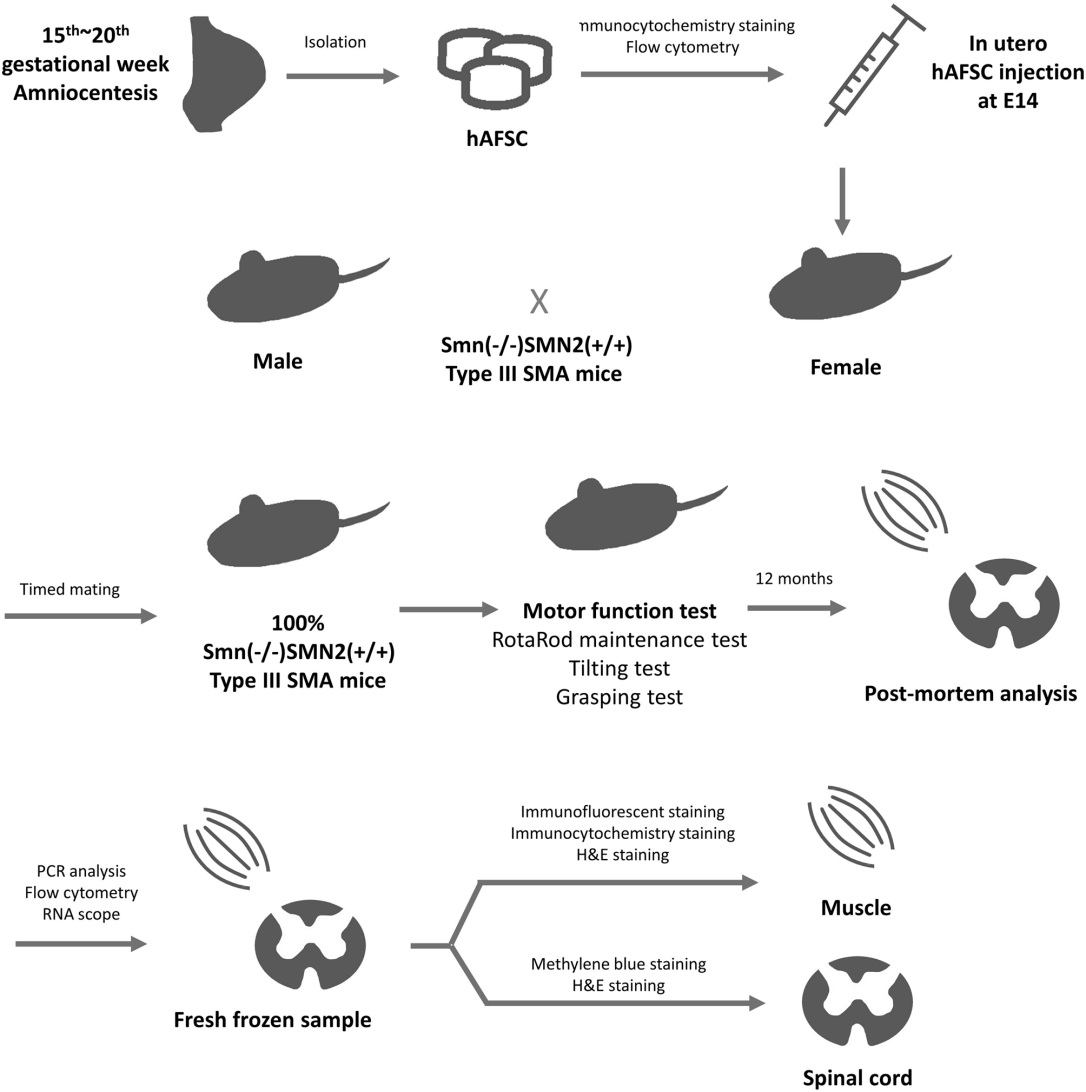
The design of experiment. Mating of male and female Smn(−/−)SMN2(+ / +) type III mice achieved 100% success. 1 × 10^5^ hAFSCs were injected in utero at embryonic day 14. *hAFSC* human amniotic fluid stem cell, *SMA* spinal muscle injury.

All 20 mice were randomly divided into two groups with or without injection of hAFSC. A total 16 SMA mice were successfully given an injection of 1 × 10^5^ hAFSC into peritoneal cavities at E14. We transplanted all hAFSC without sorting to avoid clonal section and wished to observe with minimal manipulation. A total 16 SMA mice without injection of hAFSC served as the negative control group. The hAFSC isolated from amniotic fluid served as a positive control group. All mice were born through timed mating of Smn(−/−)SMN2(+ / +) type III mice (Fig. 3). A total 15 fetuses in the treatment group (15 of 16, 87.5% survival) survived until scheduled post-mortem examination. Besides, lower survival of fetuses (10 of 16, 62.5% survival) was observed in the negative control group.

### Performance of SMA mice in three behavioral tests after hAFSC transplantation

**Figure 4:**
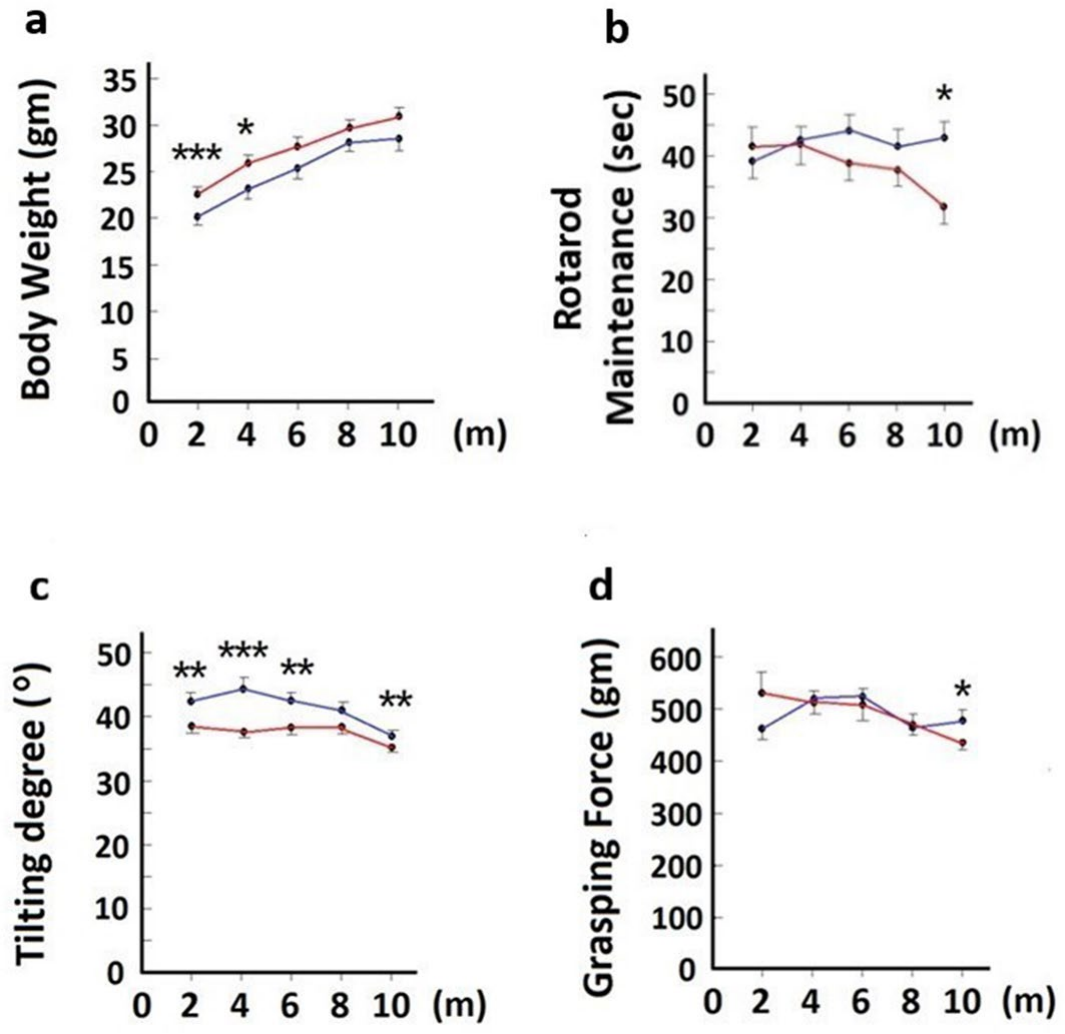
Comparison of motor ability in SMA mice with or without hAFSC transplantation. n = 16. **(a)** Body weight of SMA mice. **(b)** Rotarod maintenance time of SMA mice. **(c)** Tilting degree of wooden platform which SMA mice could hold on. **(d)** Grasping force of forearms of SMA mice. *hAFSC* human amniotic fluid stem cell; *SMA* spinal muscular atrophy. Blue line: SMA mice with hAFSC transplantation; Red line: SMA mice without hAFSC transplantation. Body weight and result of three behavioral tests were documented every two months after hAFSC transplantation. SMA mice with hAFSC transplantation has lower body weight during whole study. In all three behavioral tests, SMA mice with hAFSC transplantation has better performance during whole study.

Motor function of SMA mice was analyzed by Rotarod maintenance test, tilting test and grasping test. Body weight of each mice was documented every two months for proper evaluation. SMA mice with hAFSC transplantation has lower body weight (20.1 ± 2.30 vs 22.9 ± 1.88, [P < 0.0001]; 23.1 ± 3.15 vs 25.7 ± 2.83, [P < 0.05]) with significant difference two and four months after transplantation (Fig. 4a). In Rotarod maintenance test, SMA mice with hAFSC transplantation remained longer on the rod (42.53 ± 9.52 vs 32.07 ± 11.51, [P < 0.05]) with a significant difference ten months after transplantation (Fig. 4b). In tilting test, SMA mice with hAFSC transplantation held on the platform of higher degree (42.3 ± 3.99 vs 38.6 ± 2.68, [P < 0.01]; 44.6 ± 5.83 vs 37.8 ± 2.28, [P < 0.0001]; 42.3 ± 3.46 vs 38.5 ± 3.14, [P < 0.01]; 37.0 ± 1.95 vs 35.3 ± 1.30, [P < 0.01]) with significant difference two, four, six and ten months after transplantation (Fig. 4c). In the grasping test, SMA mice with hAFSC transplantation have a stronger grasping force of both forelimbs (482 ± 73 vs 434 ± 49, [P < 0.01]) with significant difference ten months after transplantation (Fig. 4d).

### Engraftment of hAFSC in organs of SMA mice after transplantation

**Figure 5:**
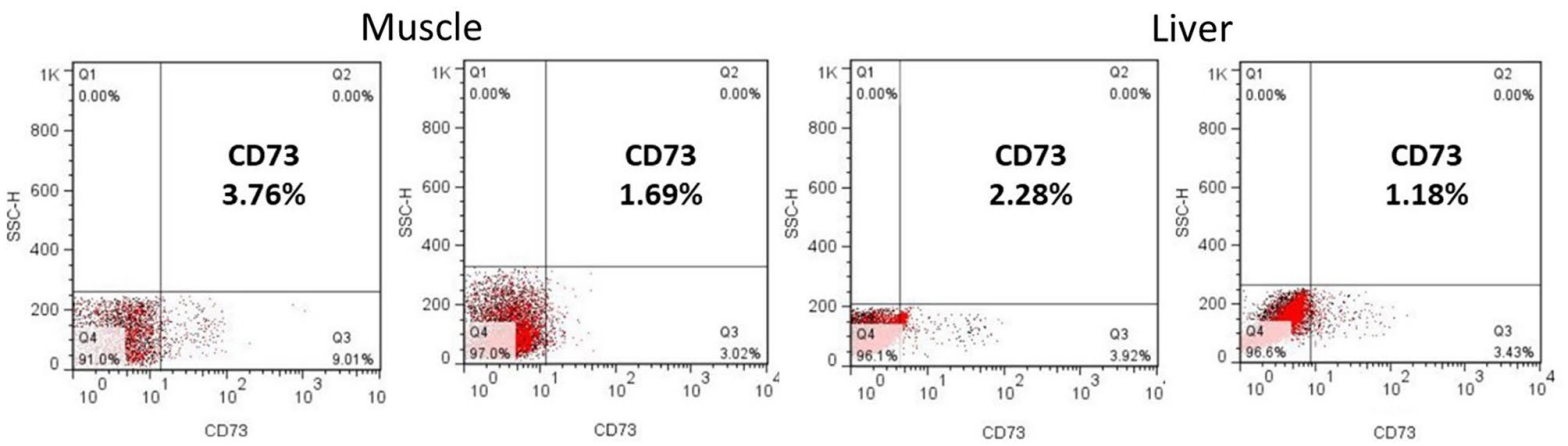
Flow cytometry analysis of muscle and liver in SMA mice after hAFSC transplantation. Muscle and liver of SMA mice with hAFSC transplantation were positive for CD73, a surface marker of MSC. Black spots indicated negative controls, and red spots indicated CD73 positives. *hAFSC* human amniotic fluid stem cell; *MSC* mesenchymal stem cell.

All organs of SMA mice were extracted for post-mortem analysis 12 months after transplantation. By flow cytometry, the muscle and liver of SMA mice with hAFSC transplantation were positive for CD73, a surface marker of MSC (Fig. 5). However, just a little signal of CD73 was detectable in the spleen, bone marrow, brain and spinal cord of SMA mice with hAFSC transplantation.

**Figure 6:**
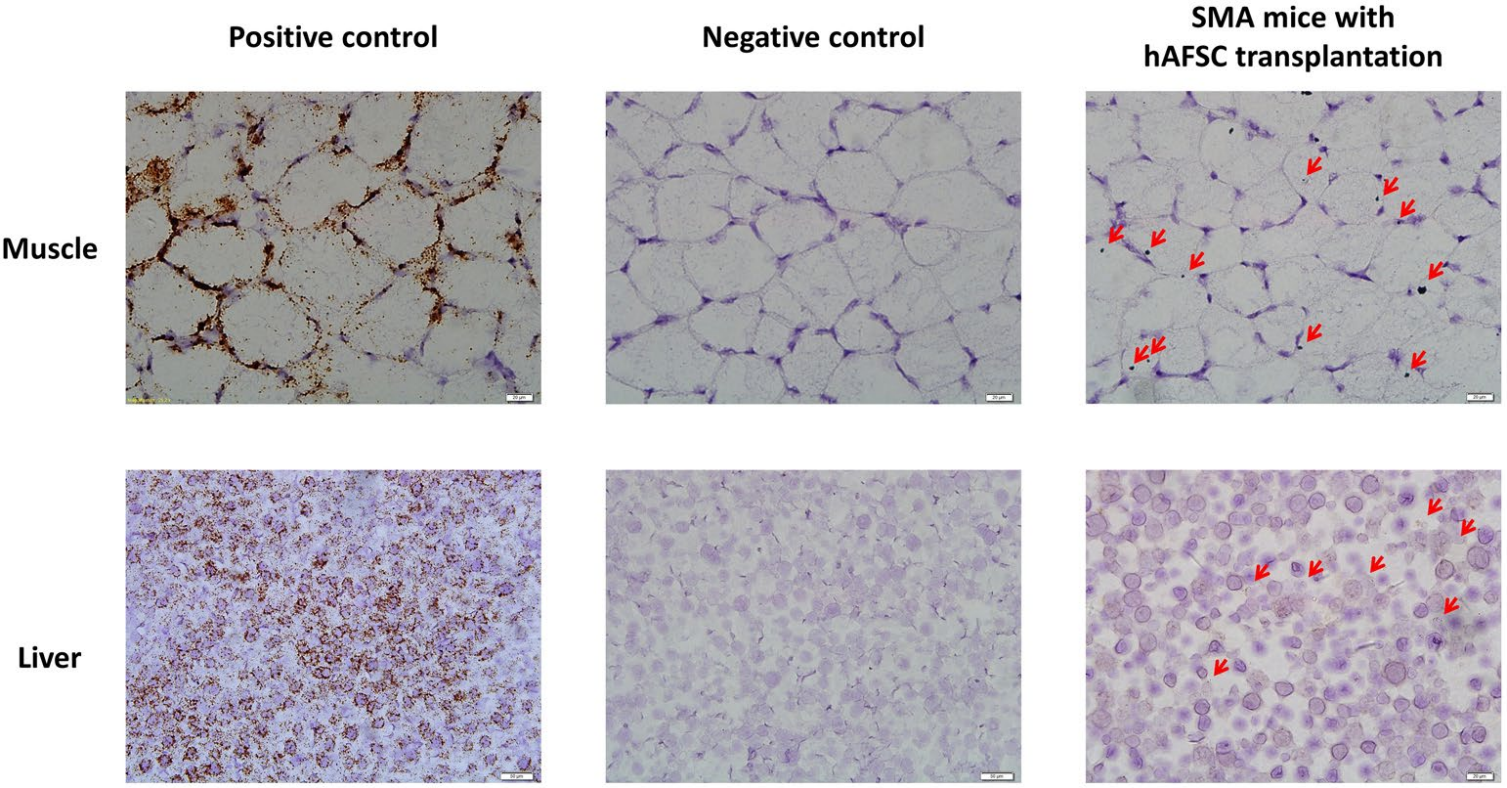
hAFSC transplantation of muscle and liver in SMA mice by RNA scope. With mouse PPIB probe, positive control SMA mice demonstrated good RNA quality. With human CD37 probe, SMA mice with hAFSC transplantation showed CD73, representing successful transplantation of human cell. *hAFSC* human amniotic fluid stem cell, *SMA* spinal muscular atrophy, *PPIB* peptidylprolyl isomerase B.

**Figure 7:**
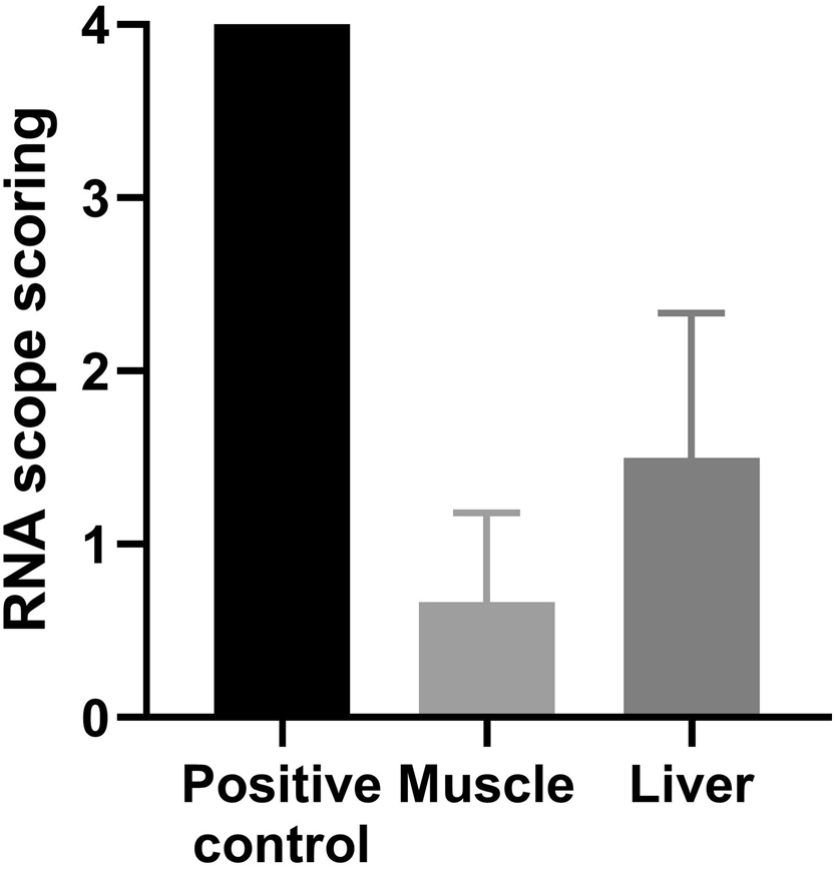
RNA scope scoring of muscle and liver from SMA mice after hAFSC transplantation. Liver expressed more CD73 than muscle. *hAFSC* human amniotic fluid stem cell.

By RNA scope, the cell of the positive control group demonstrated good RNA quality with the mouse PPIB probe (Fig. 6). Based on ACD scoring system, the semi-quantitative outcome of muscle and liver was showed (Fig. 7). The score of liver was 1.50 ± 0.88 and muscle was 0.67 ± 0.54 by RNA scope. The muscle and liver of SMA mice with hAFSC transplantation showed CD73 as an indicator of successful engraftment of the human cell. There was a higher level of engraftment in SMA mice with hAFSC transplantation compared with the negative control group. Nonetheless, little CD73 was observed in the spinal cord of SMA mice with hAFSC transplantation.

### Comparison of muscle and neuron in SMA mice after hAFSC transplantation

**Figure 8:**
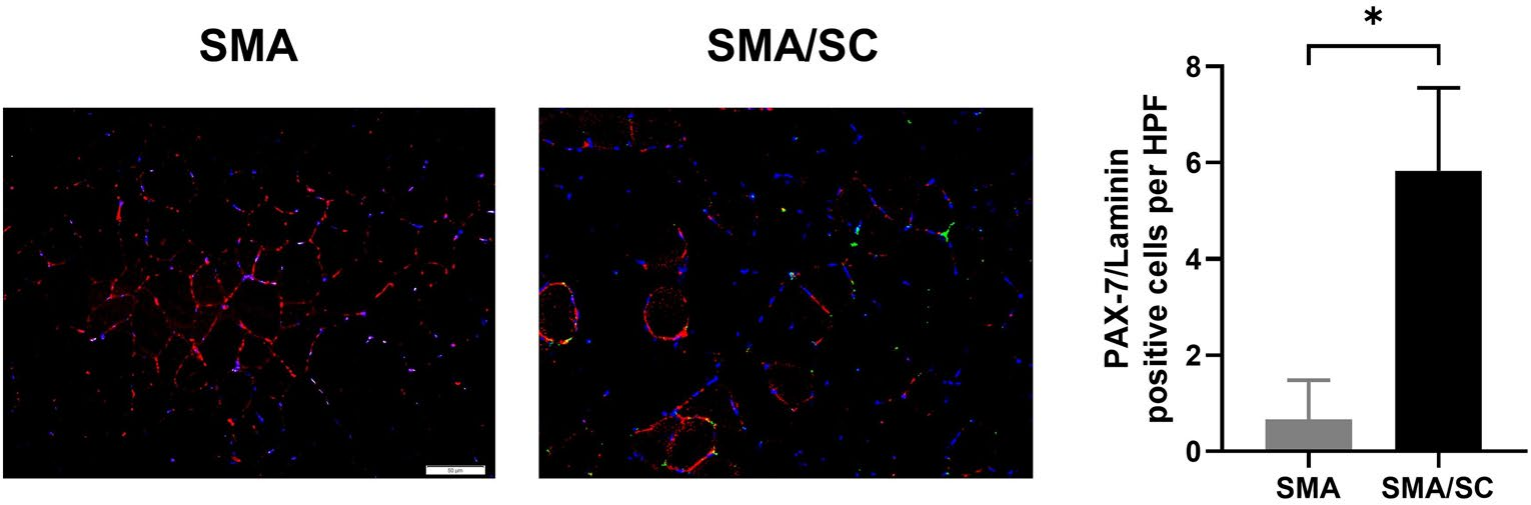
Immunofluorescent staining of muscle in SMA mice with or without hAFSC transplantation. Muscle of SMA mice with transplantation expressed Laminin (red color), PAX7 (green color) and DAPI (blue color). *HPF* high power field, *hAFSC* human amniotic fluid stem cell, *SMA* spinal muscular atrophy.

**Figure 9:**
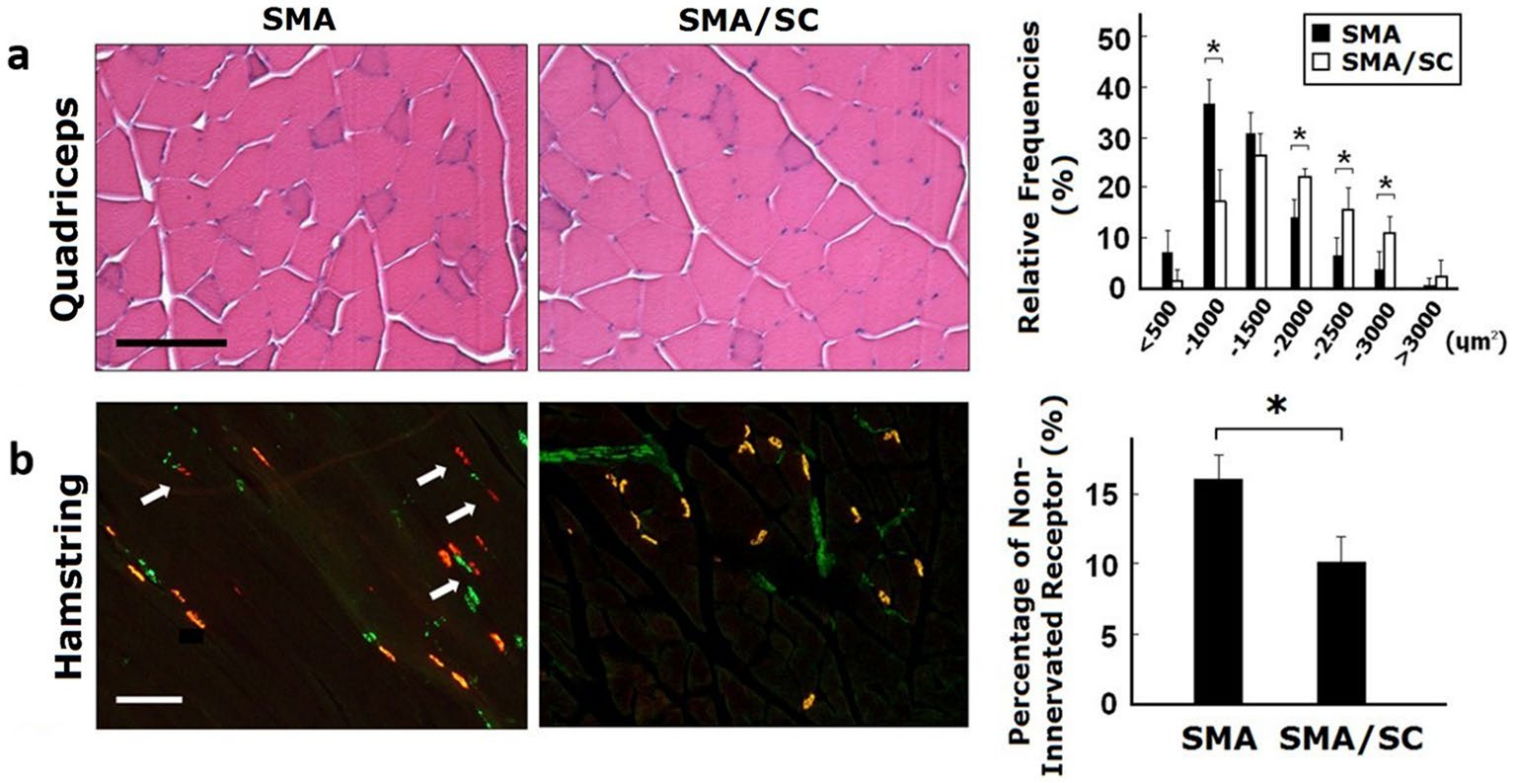
Comparison of muscle in SMA mice with or without hAFSC transplantation. n = 16. **(a)** H&E staining of tissue from quadriceps. Blue color indicated myocytes. **(b)** Immunocytochemistry staining of tissue from hamstring. Red color indicated non-innervated receptors. Higher relative frequency of myocyte and lower percentage of non-innervated receptors were detected in SMA mice with hAFSC transplantation. *hAFSC* human amniotic fluid stem cell, *SMA* spinal muscular atrophy, *SC* stem cell.

**Figure 10:**
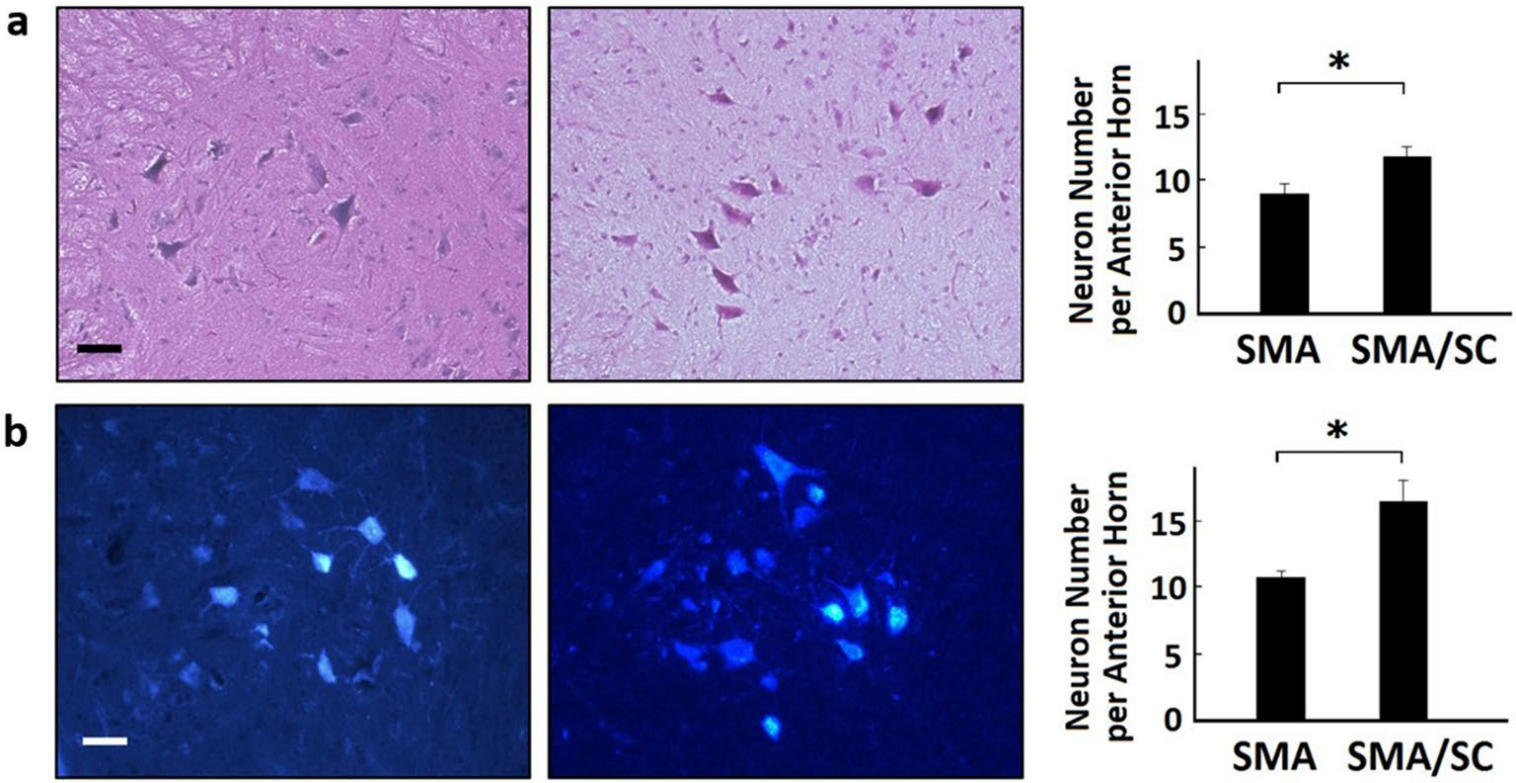
Comparison of neuron in SMA mice with or without hAFSC transplantation. n = 16. **(a)** H&E staining of tissue from spinal cord. **(b)** Methylene blue staining of tissue from spinal cord. More neurons per anterior horn were detected in SMA mice with hAFSC transplantation. *hAFSC* human amniotic fluid stem cell, *SMA* spinal muscular atrophy, *SC* stem cell.

Samples of muscle and spinal cord were made into frozen sections. The muscle of SMA mice with hAFSC transplantation expressed laminin α and PAX-7 by immunofluorescent staining (Fig. 8). PAX-7/Laminin positive cells per high power filed of SMA mice with hAFSC transplantation was significantly higher (5.83 ± 1.81 vs 0.67 ± 0.86, [P < 0.05]). Besides, hAFSC expressed IL-6 and VEGF, a cytokine and a growth factor involved in the regenerative pathway of satellite cell (Supplementary figure S1). In tissue from quadriceps, SMA mice with hAFSC transplantation demonstrated higher relative frequency of myocyte (24.3 ± 1.08 vs 15.0 ± 4.24, [P < 0.05]; 15.0 ± 2.83 vs 6.0 ± 2.83, [P < 0.05]; 10.3 ± 2.27 vs 4.0 ± 2.55, [P < 0.05]) with a significant difference in 1500–2000, 2000–2500 and 2500–3000 μm^2^ of cross-section size by H&E staining compared with the negative control group (Fig. 9a). Nonetheless, SMA mice with hAFSC transplantation demonstrated a lower relative frequency of myocyte (18.3 ± 7.08 vs 36.3 ± 4.32, [P < 0.05]) with a significant difference in 500–1000 μm^2^ of cross-section size. In tissue from the hamstring, SMA mice with hAFSC transplantation represented a lower percentage of non-innervated receptors (9.63 ± 2.71 vs 15.83 ± 2.89, [P < 0.05]) with a significant difference by immunocytochemistry staining compared with the negative control group (Fig. 9b). In tissue from the spinal cord, SMA mice with hAFSC transplantation have more neurons per anterior horn with a significant difference by both H&E and methylene blue staining (Fig. 10).

## Discussion

Due to the high carrier rate of SMA, all pregnant women should take a carrier screening test according to the recommendation by American College of Obstetrics and Gynecology^16^. Prenatal testing for SMA could be completed by amniocentesis during 15th to 20th gestational week^21^. Amniotic fluid represented not only a diagnostic specimen but also a great source of stem cells. Consequently, hAFSC were collected to establish the therapeutic potential for SMA.

To analyze the capability of differentiation and regeneration of hAFSC, the first step is to identify specific MSC markers. In our study, hAFSC showed several MSC positive markers, including CD44, CD73, CD90, CD105 and CD117. In addition, hAFSC demonstrated the pluripotency according to the expression of c-kit, a pluripotent-related marker. Identification of shared MSC markers indicated similar differentiative characteristics of other stem cells. This potential of differentiation into a trilineage-relative cell could regenerate and repair damaged tissue^22^.

Successful engraftment of hAFSC in SMA mice was the first step in transplantation. After the injection into the fetal peritoneal cavity, hAFSC would migrate widely to localize in multiple organs, such as the liver, spleen, bone marrow, brain, spinal cord and muscle. To confirm the engraftment, CD73, a surface marker of MSC, was utilized to identify the existence of a human cell in SMA mice^23^. With this stem-cell-shared characteristic, hAFSC was proved to possess the potential of differentiation and repairment. Our study showed a good distribution of hAFSC with no miscarriage related to the procedure. Flow cytometry analysis indicated a higher engraftment rate in muscle and liver. Newly generated cells with typical morphology were showed by RNA scope. However, less CD73 was detected in some hematopoietic and nervous organs, including the spleen, bone marrow and brain. Because not the entire organ contained positive cells, some samples of the same organ demonstrated a negative outcome. In addition, impaired organs, including muscle and spinal cord in SMA mice, could provide engraftment advantage to appeal more Hafsc^24^. Last but not least, cell differentiation might be incomplete due to the short interval between transplantation and post-mortem analysis. In short, the success of engraftment played a fundamental role in hAFSC transplantation.

To reduce degeneration and maintain the function of a neuron, the mechanism of hAFSC transplantation for SMA should be investigated. Declined expression of SMN protein leads to loss of motor neuron in spinal cord^25^. For example, laminin, a fundamental protein for cell differentiation, was involved in the growth of nerve axon^26^. Neural injury from SMA decreased laminin with proliferated integrin receptors to promote neural regeneration. Moreover, PAX-7, a transcription factor for neural crest development, a regulated proliferation of precursor cells in myogenesis^27^. In our study, hAFSC showed enormous PAX-7, which is also observed in SMA mice after transplantation of hAFSC. This expression of PAX-7 showed successful engraftment on organs, such as muscle and liver, in these SMA mice. Declined PAX-7 were detected in satellite cells of SMA murine model. Consistent with the previous study, SMA mice expressed scarce laminin and PAX-7^28,29^. After hAFSC transplantation, significant differential expressions of both proteins were noted by immunofluorescent staining. This result indicated that hAFSC transplantation was able to modify the RNA metabolism of neurons and muscle to deal with SMA^30^.

For further evaluation of hAFSC therapy for SMA, successful engraftment should be identified at the cellular level. Neural dysfunction of SMA was derived from the reduction of motor neurons and neuromuscular junctions^8^. In line with the previous study, SMA mice represented fewer myocytes of muscle and fewer neurons of anterior horn^31^. After hAFSC transplantation, the number of both cells increased significantly. Newly generated cells demonstrated typical morphology by H&E staining. Additionally, a lower percentage of non-innervated receptors in SMA mice with hAFSC transplantation suggested improved integrity and survival of the entire motor unit. This outcome indicated hAFSC provided recovery function for neural injury of SMA. From a clinical aspect, three behavioral tests were conducted to analyze practical effectiveness of hAFSC therapy. In the previous study, SMA mice performed poorly in all Rotarod maintenance test, tilting test and grasping test^32–34^. After hAFSC transplantation, muscle power of maintaining, holding and grasping improved significantly in our study. Though these behavioral tests might be affected by body weight, rostro-caudal axis, and etc., these favorable results still showed the therapeutic potential of hAFSC transplantation for SMA.

Our data represented the usefulness of SMA mice as a model to study hAFSC therapy. However, there were still a few limitations in this study. First, low level of tissue engraftment was noted due to the small number of hAFSC transplantation. Nevertheless, only 1% level of protein expression was needed to improve some congenital diseases^35^. Therefore, the level of engraftment we achieved might be sufficient to deal with SMA in our study. Increasing the number of hAFSC would be a choice to elevate the level of engraftment. Nonetheless, the expansion of hAFSC leads to the proliferation of committed MSC progenitor at an inappropriate location. Secondly, this study lacked data of maternal and fetal immune response because we focused on the presentation of engraftment. Data of immune response in comparing allogenic and maternal human-leukocyte-antigen-matched hAFSC transplantation was necessary to investigate the immune rejection of hAFSC. Thirdly, intraperitoneal injection of hAFSC is the optimal route for transplantation. Direct injection might do harm to the spinal cord. Based on the capability of migration, hAFSC could engraft on target lesion and repair damaged tissue. In addition, multiple injections of hAFSC were allowed due to its inherent safety. However, this intervention remained a danger for the fetus in the first trimester. Improvement of fetal imaging would make hAFSC transplantation more feasible.

SMA impaired motor neuron during embryogenesis and reduced acquisition of myelinated axon after birth. In-utero treatment was required to prevent irreversible degeneration^36^. Prenatal hAFSC therapy preserved the time window to treat SMA at an early stage. In our study, hAFSC transplantation showed the potential of neurogenesis. Vascular endothelial growth factor, an angiogenetic factor, from hAFSC was crucial for these two modifications of the nervous system^37^. Its paracrine effect enhanced the repairing in neural injury from SMA^38^. The role of VEGF in hAFSC transplantation and combined therapy with antisense oligonucleotide could be further studied in the future^39^.

## Conclusion

SMA does irreversible harm to the fetus in early gestation. This study demonstrated the therapeutic potential of prenatal hAFSC transplantation for SMA. By amniocentesis in the regular prenatal examination, hAFSC of a small number are collected. hAFSC therapy preserves the time window to treat in the uterus. To treat SMA, this therapy could be applied with antisense oligonucleotide in the future.

## Methods

### Mice

SMA model mice were generated by deletion of exon 7 of (Survival motor neuron) Smn gene and knock-in of human *SMN2* (*Smn*^*−/−*^*SMN2*^+*/−*^)^40^. To obtain a homogenous genetic background, variant mice presented with more severe diseases were reproduced through back-crosses. Another milder type of SMA model mice represented two alleles of SMN2 transgenes (*Smn*^*−/−*^*SMN2*^+*/*+^). Each allele consisted of two copies of SMN2 transgene. Genotypes were confirmed by PCR analysis. This study was approved by the Institutional Animal Care and Use Committee of Chang Gung Memorial Hospital (IACUC Approval No. 2012121908 and No. 2014121714). All procedures were in accordance with the relevant guidelines and regulations of this committee. Mice were under the care of the laboratory animal center of National Taiwan University College of Medicine and supplied with sterile water and rodent pellets ad libitum. The authors had read the ARRIVE guidelines, and the study was conducted according to it.

### Isolation and characterization of hAFSC

hAFSC was obtained from freshly collected amniotic fluid of normal 46, XX karyotype by routine amniocentesis from healthy pregnant donors at 15th–20th gestational week^41^. The study was performed in line with the principle of Declaration of Helsinki. All procedures were approved by Chang Gung Medical Foundation Institutional Review Board (CGMF Ref. No. 102-1994B and No. 103-7081B). Written informed consent was obtained from all eligible participants. These cells in suspension were implanted in TPP Tissue Culture Petri Dishes (TPP, Switzerland). The amniotic culture medium is composed of Chang medium B and Chang medium C (Irvine Scientific, USA), MEM Alpha solution (63%, Life Technology, UK), fetal bovine serum (15%, Invitrogen, UK), antibiotics (penicillin and streptomycin) and L-glutamine (1%, Invitrogen, UK). The dishes were incubated in an incubator that was constantly under 37ºC, 95% air, and 5% carbon dioxide. Once 60–80% confluence was achieved, the adherent cells were detached from the plate by 0.05% trypsin and 0.02% sodium-EDTA (Life Technologies, UK). Then, they were transferred into a bigger dish or dispensed evenly into 2 dishes.

The specific surface antigens of hAFSC were characterized by flow cytometry analysis. The cells were stained with phycoerythrin (PE)—conjugated antibodies against CD44, CD73, CD90, CD105, and CD45 (BD PharMingen, CA) and protected from light for 30 min at 4 ˚C. Following incubation, the cells were washed twice with Phosphate Buffered Saline (PBS). Thereafter, the cells were analyzed with the Calibur flow cytometer (Becton Dickinson, Heidelberg, Germany).

### Immunocytochemistry staining

To confirm the identity of stem cells, hFASC with specific surface markers would demonstrate green fluorescent protein by immunocytochemistry staining (ICC)^18^. 1 × 10^5^ hAFSC were seeded on sterilized glass coverslips which were placed in six-well tissue culture plates and incubated at 37 °C in a humidified CO_2_ incubator overnight. The culture medium was aspirated from each well and fixed by 4% paraformaldehyde for 20 min at room temperature (RT). Then, the cells were permeabilized by 0.2% Triton X-100 in PBS for 5 min at RT and washed three times with PBS. Blocked with 5% fetal bovine serum in PBS for 30 min at RT, the cells were incubated in a humidified chamber overnight at 4 °C after appropriate dilution of human PAX-7 (LifeSpan BioSciences) to the glass coverslips. Eventually, the cells were stained with 546 anti-rabbit IgG antibodies (Santa Cruz Biotechnology) and DAPI for one hour. All images were detected by confocal microscopes (Leica,Wetzlar, Germany).

### Transplantation

After the sixth passage, hAFSC were collected and prepared to a final concentration of 1 × 10^5^ cells per 10 μL in PBS^42^. On embryonic day 14 (E14) after the timed mating, pregnant mice would be sedated by vaporized Isoflurane-Vet 5% in oxygen (VetTech Solutions Ltd, UK) and maintained by vaporized Isoflurane-Vet 2% in oxygen. The belly was sterilized by Povidone-Iodine surgical scrub (Vetasept, UK). Via longitudinal laparotomy, the abdominal cavity was exposed and the fetal mice within the bicornuate uterus were counted. 1 × 10^5^ hAFSC (treatment group) or PBS (control group) will be injected through the maternal uterus and the surrounding AF into the peritoneal cavity of each pup via small-gauge RN needles (27–33 gauge, Hamilton). The pregnant mice will be checked by the caretaker in the animal center every day, and most of them delivered these recipient mice on E20 (six days after transplantation). The new-born mice were sacrificed by CO_2_ inhalation 12 months after the delivery. The postmortem analysis was performed to see the evidence of human cell engraftment.

### Motor function tests

Three behavioral tests were used to evaluate the motor function of mice two months after birth^43^. In the Rotarod maintenance test, the mice were placed on a Rotarod, while the rolling rate of the transverse rod increased and the time each mouse remained on the rod was recorded. In the tilting test, the mice were placed on a progressively tilting wooden platform. The highest degree of inclination at which the mice could hold on for five seconds was recorded. For the grasping test, the grasping force of both forelimbs was tested using the grasping machine. Each mouse underwent ten studies every two months and the five best results were averaged.

### Detection of human cells by DNA extraction and PCR analysis

All organs including heart, liver, kidney, lung, spleen, muscle, bone marrow, brain and spinal cord of the SMA Type III mice were extracted^44^. Genomic DNA was obtained from the tissues by ZR Genomic DNA-Tissue MiniPrep kit (ZYMO RESEARCH, Orange, CA). The DNA concentration was determined by optical density with a ASP-3700-Micro-volume spectrophotometer (ACTGene, USA) and then conducted by standard DNA PCR analysis (AmpliTaq GoldR 360 Master Mix, MyCycler Thermal Cycler, Bio-Rad, CA, USA). To check the presence of hAFSC in the murine background, we used the primers for two ubiquitous genes by PCR. Primers specific for the human β2-microglobulin gene were utilized to amplified human sequences (huβ2Micro: 5′CAGGTTTACTCACGTCATCCAGC3′; huβ2Micro: 5′TCACATGGTTCACACGGCAGGC3′). For positive control, we used the housekeeping gene β-actin primers that amplified both human and murine DNA (β-actin: 5′GTGACGAGGCCCA GAGCAAGAG3′; β-actin: 5′ACGCAGCTCATTGTAGAAGGTGT GG3′). PCR condition was 95 °C × one minute for one cycle followed by 95 °C × 30 s/57.1 °C × 1 min/72 °C × 30 s for 42 cycles. For negative controls, DNA from non-injected mice was used. For positive controls, DNA from hAFSC was used. The 235-base pair (bp) amplified fragments of human β2 microglobulin or 122 bp β-actin were separated on 2% agarose gels.

### Flow cytometry

Freshly mice liver, muscle, spleen, brain, spinal cord and bone marrow were transformed into homogenate through a 40 micro cell strainer into a 50 ml conical tube^45^. Those cells were washed once and treated in the pellet with 1 × RBC lysis buffer (Gibco, Life technologies) 2 ml for 1 min at RT, and they were washed again. Surface markers stained with PE-conjugated anti-human CD73 (eBioscience) antibody for 30 min at 4 °C in the dark. The cells were washed twice with PBS in 3 ml and analyzed by flow cytometry (Becton Dickinson, Heidelberg, Germany).

### Immunofluorescent staining

To observe the change of muscle, specific proteins were under screening by Immunofluorescent staining^42^. SMA Type III mice liver, muscle and spinal cord were fixed as frozen sections slide with pre-cooled acetone for 10 min, then washed in PBS 3 times for five minutes. The slides were blocked with 10% fetal bovine serum in PBS for 20 min at RT. Diluted mouse laminin α (Santa Cruz Biotechnology) was applied to the muscle, and human PAX-7 (LifeSpan BioSciences) was applied to the liver, muscle, spinal cord slides. The slides were incubated in a humidified chamber overnight at 4 °C and stained with 546 anti-rabbit IgG antibodies (Santa Cruz Biotechnology) for 45 min and then DAPI. All images were detected by confocal microscopes (Leica, Wetzlar, Germany).

### RNA scope

Human and pig AFSC were stained as a positive and negative control. The cells were seeded on slides and fixed with 4% paroformaldehyde (PFA) for 60 min, followed by pretreating 1 (H2O2 block) at RT for 10 minutes^46^. The slides were placed in 100% EtOH for 5 min at RT and dried in air for 5 min at RT. The Immedge hydrophobic barrier pen was utilized to draw a barrier 2–4 times around each section. The slides were incubated with pretreat 3 for 20 min at RT for protein digestion and rinsed twice in dH2O. Target probe human CD73 was hybridized for two hours at 40 °C in HybEZ oven. The slides were incubated with Amp1 (preamplifier) for 30 min at 40 °C , Amp2 (background reducer) for 15 min at 40 °C, Amp3 (amplifier) for 30 min at 40 °C, Amp4 (label probe) for 15 min at 40 °C in HybEZ oven, Amp5 for 30 min and Amp6 for 15 min at RT. After all incubation steps, the slides were rinsed in 1 × Wash buffer for 2 × 2 min at RT. Chromogenic detection was performed with DAB followed by counterstaining with hematoxylin (American MasterTech Scientific, Lodi, CA).

### Fresh frozen sample

Liver, Muscle, Spinal Cord was embedded in optimum cutting temperature compound (OCT, Leica) and sectioned at − 25 °C with a thickness of 15 µm and mounted on slides^47^. Fix the sections by 4% PFA for 15 min at 4 °C. Dehydrate the sections with 50%, 70%, 100% EtOH at RT for five minutes. Use the Immedge hydrophobic barrier pen to draw a barrier 2–4 times around each section. Place the sections in pretreating 1 at RT for 10 min, followed by pretreating 4 at RT for 30 min. Target probes human CD73, PPIB (positive control) and DapB (negative control) were then hybridized for 2 h at 40 °C in HybEZ oven. Preamplifier, amplifier, and chromogenic detection were as described above for cultured cells.

### H&E staining

Frozen tissue maximal sections from the spinal cord (10-μm thickness) and quadriceps muscle (5 μm) were stained in hematoxylin and eosin (H&E)^48^. Neurons with a size greater than 200 µm^2^ located in the anterior horn of the spinal cord were analyzed. We counted the number of neurons (only neurons showing nuclei) according to their cell size: 200–400, 400–600 and > 600 μm^2^, respectively. The size of myocytes from the quadriceps was analyzed. We calculated the number of myocytes according to their cross-section size.

### Immunohistochemistry staining

The hamstring muscle was removed at the age of 12 months. The maximal section of tissue was placed in 4% paraformaldehyde at RT for 3 h and transferred to 15% sucrose for 1 day and 30% sucrose at 4 °C for another 3 days^49^. Then, the specimen was rapidly frozen in liquid nitrogen-cooled isopentane. Frozen serial sections of muscles were cut into 10 μm thickness and blocked with 5% serum. The sections were incubated overnight at 4 °C with anti-neurofilament 200 kDa (1:200; Millipore) or α-bungarotoxin Alexo Fluor 555 conjugate (1:1000; Invitrogen). The samples were washed with TBS-T (10 mM Tris–HCl, pH 8.0, 150 mM NaCl, and 0.1% Tween 20) and incubated for two hours at RT with the appropriate fluorescence-conjugated secondary antibodies (1:200; Molecular Probes, Eugene, OR). Finally, the tissues were examined with a laser-scanning confocal microscope (Leica TCS SP2 Spectral Confocal System, Leica Microsystems, Mannheim, Germany).

### Retrograde tracing

For retrograde tracing in motor neurons, 8 μl of fluorogold (Fluorochrome, Denver, CO) was injected into bilateral gastrocnemius and tibialis anterior muscles of mice (2 μl for each muscle) at the age of 12 months with a 30-gauge needle^50^. Mice were sacrificed 5 days after the injection, and their spinal cord was removed. After paraformaldehyde fixation, frozen serial sections of the whole lumbar segment of the spinal cord were cut at 10-μm thickness and processed for histological analysis.

### Statistical analysis

All mice were born through timed mating of Smn(−/−)SMN2(+ / +) type III mice. 100% success of SMA model mice reproduction was expected. Therefore, we prepared 20 female mice and evenly divided them into two groups with or without injection of hAFSC to obtain as many fetuses as possible.

The performance of three motor function test was analyzed by Student’s t test**.** Numbers of muscle, neuron and neuromuscular junctions were analyzed by Student’s t test. Prism (version 6) was utilized for all statistical analysis. While P was less than 0.05, null hypothesis was rejected.

## Supplementary Information

Supplementary Figure S1.
