# Supplementary material for: Prenatal transplantation of human amniotic fluid stem cell could improve clinical outcome of type III spinal muscular atrophy in mice
Source: Sci Rep. 2021 Apr 28;11:9158. doi: 10.1038/s41598-021-88559-z (PMC8080644; doi:10.1038/s41598-021-88559-z)

**Supplementary figure 1** Immunofluorescent staining of hAFSC. hAFSC expressed PAX7 (green color), VEGF (red color) and IL-6 (green color). hAFSC: Human amniotic fluid stem cell


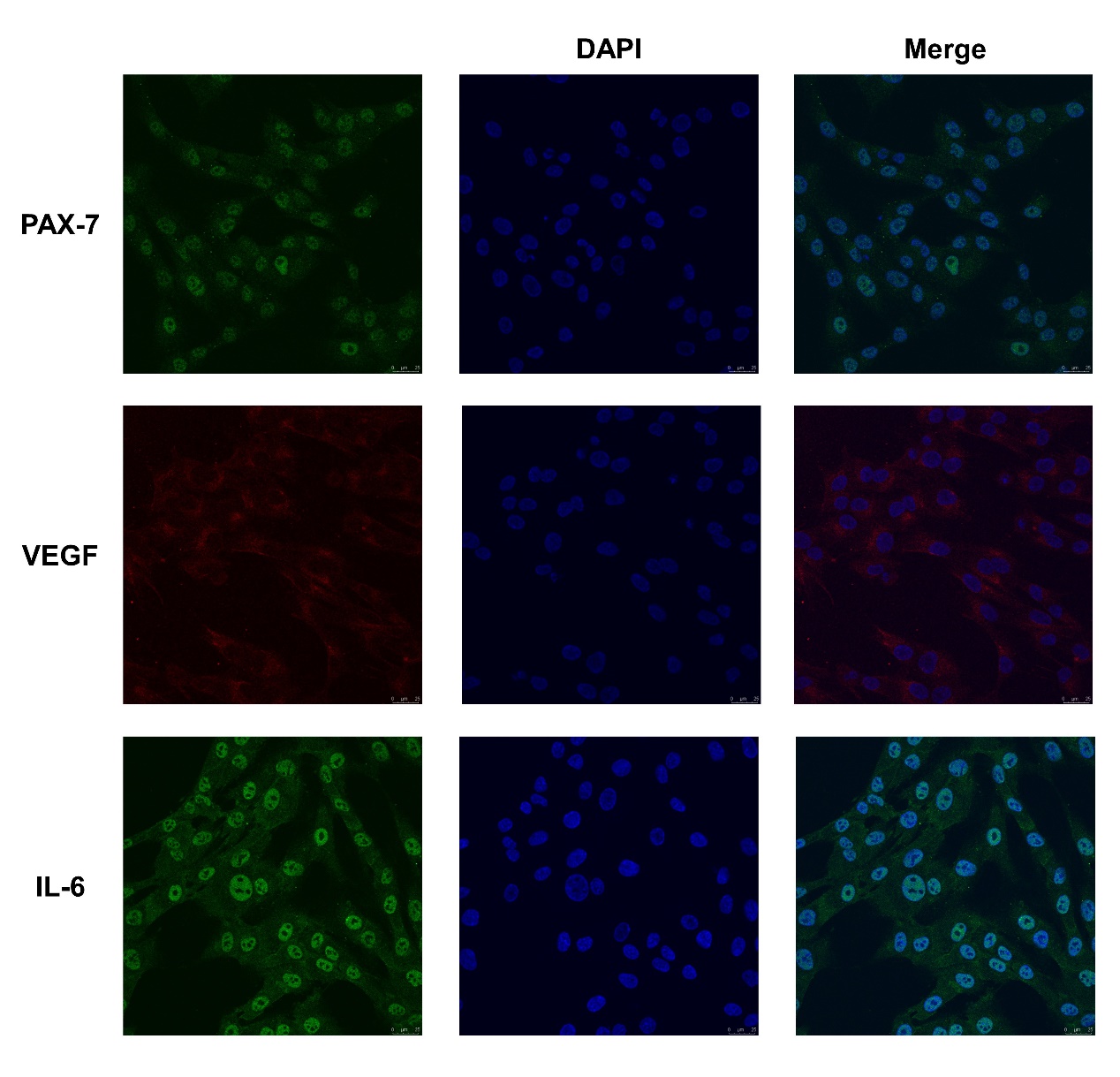

Supplement: Supplementary file 1 — Supplementary Figure S1. [file 41598_2021_88559_MOESM1_ESM.docx]
